# Phosphorylation of Rab37 by protein kinase C alpha inhibits the exocytosis function and metastasis suppression activity of Rab37

**DOI:** 10.18632/oncotarget.20998

**Published:** 2017-09-18

**Authors:** Hong-Tai Tzeng, Tsung-Hsin Li, Yen-An Tang, Chung-Han Tsai, Pei-Jung Frank Lu, Wu-Wei Lai, Chi-Wu Chiang, Yi-Ching Wang

**Affiliations:** ^1^ Department of Pharmacology, College of Medicine, National Cheng Kung University, Tainan, Taiwan; ^2^ Cancer Therapeutics and Stratified Oncology, Genome Institute of Singapore, Agency for Science, Technology and Research (A*STAR), Singapore; ^3^ Institute of Basic Medical Sciences, College of Medicine, National Cheng Kung University, Tainan, Taiwan; ^4^ Institute of Clinical Medicine, College of Medicine, National Cheng Kung University, Tainan, Taiwan; ^5^ Division of Thoracic Surgery, Department of Surgery, National Cheng Kung University Hospital, College of Medicine, National Cheng Kung University, Tainan, Taiwan; ^6^ Institute of Molecular Medicine, College of Medicine, National Cheng Kung University, Tainan, Taiwan

**Keywords:** Rab37, PKCα, exocytosis, metastasis, prognosis

## Abstract

We previously identified a novel Rab small GTPase protein, Rab37, which plays a critical role in regulating exocytosis of secreted glycoproteins, tissue inhibitor of metalloproteinases 1 (TIMP1) to suppress lung cancer metastasis. Patients with preserved Rab37 protein expression were associated with better prognosis. However, a significant number of the patients with preserved Rab37 expression showed poor survival. In addition, the molecular mechanism for the regulation of Rab37-mediated exocytosis remained to be further identified. Therefore, we investigated the molecular mechanism underlying the dysregulation of Rab37-mediated exocytosis and metastasis suppression. Here, we report a novel mechanism for Rab37 inactivation by phosphorylation. Lung cancer patients with preserved Rab37, low TIMP1, and high PKCα expression profile correlate with worse progression-free survival examined by Kaplan-Meier survival, suggesting that PKCα overexpression leads to dysfunction of Rab37. This PKCα-Rab37-TIMP1 expression profile predicts the poor outcome by multivariate Cox regression analysis. We also show that Rab37 is phosphorylated by protein kinase Cα (PKCα) at threonine 172 (T172), leading to attenuation of its GTP-bound state, and impairment of the Rab37-mediated exocytosis of TIMP1, and thus reduces its suppression activity on lung cancer cell motility. We further demonstrate that PKCα reduces vesicle colocalization of Rab37 and TIMP1, and therefore inhibits Rab37-mediated TIMP1 trafficking. Moreover, Phospho-mimetic aspartate substitution mutant T172D of Rab37 significantly promotes tumor metastasis *in vivo*. Our findings reveal a novel regulation of Rab37 activity by PKCα-mediated phosphorylation which inhibits exocytic transport of TIMP1 and thereby enhances lung tumor metastasis.

## INTRODUCTION

Rab small GTPases consist of the largest subgroup of Ras superfamily and function as regulators of intracellular vesicle transport, such as endocytosis, receptor recycling and exocytosis [1–3]. Crosstalk between complex networks of Rab and effector proteins, including kinases, phosphatases, sorting or tethering adaptors, and motors ensures precise regulation of vesicle traffic [4–6]. All Rab GTPases have consensus motifs to bind GDP (inactive form) and switch to active form when bound to GTP [7]. Notably, emerging evidence show that dysregulated Rab-mediated transport pathways and aberrant expression of Rab GTPases are implicated in tumorigenesis. Indeed, overexpression of Rab2A, Rab3D, Rab8, Rab11, Rab21, Rab23, Rab25, Rab27B and Rab35 promotes tumor cell migration and invasion [8–16].

We have previously cloned human *Rab37* and found that *Rab37* mRNA was down-regulated by promoter hypermethylation in non-small cell lung cancer patients. The low level of *Rab37* mRNA expression was associated with tumor metastasis [17]. Therefore, we hypothesize that Rab37 acts as a tumor suppressor in lung cancer. We have identified TIMP1 and TSP1 as the potential trafficking cargos of Rab37 by secretome analysis [18]. We further demonstrate that expression of Rab37 suppresses tumor progression by mediating exocytosis of TIMP1 and TSP1 to inactivate extracellular metalloproteinase 9 and to inhibit tumor neovasculature respectively [18, 19]. Since the sophisticated regulation of Rab activity is important for spatiotemporal control of vesicle traffic, it remains elusive whether phosphorylation modification of Rab37 regulates its functions in exocytosis and tumorigenesis. In this report, we show that Rab37 phosphorylation by PKCα inactivates Rab37-mediated exocytic transport by decreasing GTP-bound active form and thereby enhances lung tumor invasion and metastasis.

## RESULTS

### Clinical impacts of inverse correlation of PKCα/TIMP1 expression

**Figure 1:**
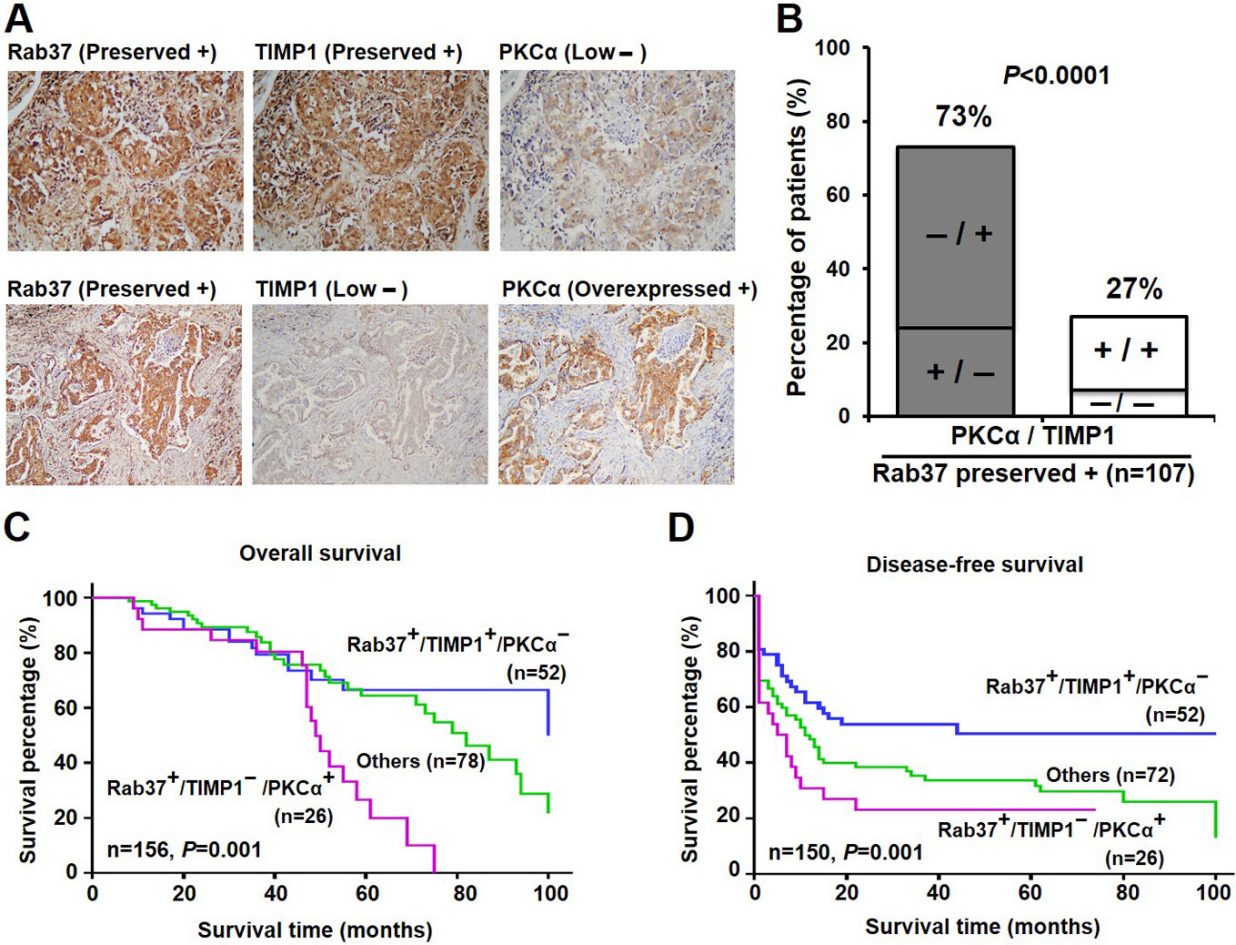
High level of PKCα associated with poor survival in the lung cancer patients with preserved expression of Rab37 and low level of TIMP1 (**A**) Immunohistochemistry analysis of Rab37, TIMP1 and PKCα expression in lung specimen from two lung cancer patients. (**B**) Concordance analysis of TIMP1 (+, preserved; -, low expression) and PKCα (+, overexpression; -, low expression) in lung cancer patients with preserved Rab37 expression (*n =* 107). The percentage of discordant group (gray bar) and concordant group (white bar) was indicated in the plot. *P*-value was calculated by Pearson χ^2^-test. (**C** and **D**). Overall survival and disease-free survival of Kaplan-Meier analysis were shown. The normal expression of Rab37 and TIMP1, overexpressed PKCα level were designated as “+”; and low expression of indicated proteins as “-”. *P*-values were determined by log-rank test. Lung cancer patients with expression profile of normal Rab37, low TIMP1 and overexpressed PKCα showed worse overall survival (C) and disease-free survival (D).

We previously reported that preserved expressions of metastatic suppressor Rab37 and its cargo, TIMP1, were associated with better prognosis [18]. However, a subgroup of lung cancer patients with preserved Rab37 level was linked to high mortality. Therefore, we hypothesized that the patients with poor prognosis but preserved Rab37 expression may result from the dysfunction of Rab37 in their tumor tissues. Since members of PKC family regulate physiological function of some Rab GTPases by phosphorylation [20, 21], a panel of PKC inhibitors was used to screen for kinase of Rab37 (Supplementary Figure 1A). Additional gene manipulation experiments showed that PKCα was the candidate kinase for Rab37 phosphorylation (see below). We therefore tested whether Rab37 dysregulation was associated with PKCα level in clinical lung cancer specimens. We examined Rab37, TIMP1 and PKCα expression patterns in tumor specimens from 158 lung cancer patients and assigned protein expression levels into preserved, overexpressed and low expression by immunohistochemical analysis (Figure 1A). A total of 107 (67.7%) patients showed preserved Rab37 protein expression (Figure 1B and Table 1). We then performed stratification analyses for TIMP1 and PKCα expression in this subgroup of 107 patients with preserved Rab37 expression. Strikingly, we observed a strong inverse correlation between TIMP1 and PKCα (73%, *p* < 0.0001; Figure 1B and Table 1). Patients with preserved Rab37 expression and low TIMP1 expression were significantly associated with PKCα overexpression, supporting our hypothesis that PKCα overexpression leads to dysfunction of Rab37.

**Table 1:** Clinicopathological parameters in lung cancer patients enrolled in the present study

Characteristics	Total	Rab37 expression			TIMP1 expression			PKCα expression	
		Low (%)	Preserved (%)	Low (%)		Preserved (%)	Overexpressed (%)		Low (%)
	*N* = 158	51 (32.3%)	107 (67.7%)	72 (45.6%)		86 (54.4%)	85 (53.8%)		73 (46.2%)
**Type**									
SCC	21	9 (42.9%)	12 (57.1%)	17 (81.0%)		4 (19.0%)	16 (76.2%)		5 (23.8%)
ADC	137	42 (30.7%)	95 (69.3%)	55 (40.1%)		82 (59.9%)^0.001^	69 (50.4%)		68 (49.6%)^0.027^
**Stage**									
Stage I–II	107	27 (25.2%)	80 (74.8%)	47 (43.9%)		60 (56.1%)	58 (54.2%)		49 (45.8%)
Stage III–IV	51	24 (47.0%)	27 (53.0%)^0.009^	25 (49.0%)		26 (51.0%)	27 (53.0%)		24 (47.0%)
**T stage**									
Stage 1–2	127	38 (29.9%)	89 (70.1%)	53 (41.7%)		74 (58.3%)	68 (53.5%)		59 (46.5%)
Stage 3–4	29	12 (41.4%)	17 (58.6%)	18 (62.1%)		11 (37.9%)^0.047^	17 (58.6%)		12 (41.4%)
**N stage**									
N0	88	23 (26.1%)	65 (73.9%)	41 (46.6%)		47 (53.4%)	51 (58.0%)		37 (42.0%)
> N1	69	27 (39.1%)	42 (60.9%)	30 (43.5%)		39 (56.5%)	34 (49.3%)		35 (50.7%)
**M stage**									
M0	133	38 (28.6%)	95 (71.4%)	58 (43.6%)		75 (56.4%)	66 (49.6%)		67 (50.4%)
> M1	23	12 (52.2%)	11 (47.8%)^0.025^	13 (56.5%)		10 (43.5%)	19 (82.6%)		4 (17.4%)^0.003^
**TIMP1 expression**									
Low	72	38 (52.8%)	34 (47.2%)	--		--	54 (63.5%)		18 (24.7%)
Preserved	86	13 (15.1%)	73 (84.9%)^0.001^	--		--	31 (36.5%)		55 (75.3%)^0.001^

NOTE: SCC, squamous cell carcinoma; ADC, adenocarcinoma; --, not applicable.

The data was analyzed by Pearson χ^2^ test. *P* values with statistical significance are as indicated.

To determine the prognostic effects of Rab37, TIMP1 and PKCα expression, survival curves were generated using the Kaplan-Meier method. Patients with preserved Rab37 and TIMP1 expression, and low PKCα expression had better overall survival and disease-free survival, while patients with preserved Rab37, low TIMP1, and high PKCα displayed significantly worse outcome (*P* = 0.001 for overall survival; *P* = 0.001 for disease-free survival, Figure 1C and 1D). We then performed univariate and multivariate Cox regression analyses to determine whether preserved Rab37, low TIMP1 and high PKCα expression pattern is an independent prognostic factor. The univariate Cox regression analysis revealed that patients with preserved Rab37, low TIMP1 and PKCα overexpression, with late stage, larger tumor size status, lymph node metastases or distant organs metastases had an increased risk for poor outcome (Table 2). Remarkably, multivariate Cox regression analysis by including all variables with a significant association in univariate analysis showed that patients with preserved Rab37, low TIMP1 and high PKCα expression pattern correlated with a relative risk of death of 3.81 (*P* = 0.002, confidence interval = 1.66–8.72; Table 2), suggesting that preserved Rab37, low TIMP1 and high PKCα expression was an independent risk factor of poor outcome. Taken together, these clinical results link PKCα overexpression with poor clinical outcome even in the group of patients with preserved expression of metastatic suppressor Rab37.

**Table 2:** Cox regression analysis of risk factors for cancer-related death in lung cancer patients

Characteristics	Univariate analysis		Multivariate analysis	
	HR (95% CI)	*P*-value^a^	HR (95% CI)	*P*-value^a^
**Rab37/TIMP1/PKCα** **expression**				
+/+/−	1.00		1.00	
Others	1.27 (0.68–2.38)	0.450	1.53 (0.78–3.01)	0.215
+/−/+	3.15 (1.55–6.38)	**0.001**	3.81 (1.66–8.72)	**0.002**
**Type**				
ADC	1.00		--^b^	
SCC	1.24 (0.35–1.41)	0.283	--^b^	--^b^
**Stage**				
Stage I-II	1.00		1.00	
Stage III-IV	1.73 (1.05–2.87)	**0.032**	1.28 (0.64–2.57)	0.488
**T stage**				
Stage 1–2	1.00		1.00	
Stage 3–4	1.89 (1.08–3.31)	**0.026**	1.07 (0.54–2.11)	0.848
**N stage**				
N0	1.00		1.00	
> N1	1.83 (1.09–3.07)	**0.022**	1.75 (0.87–3.49)	0.115
**M stage**				
M0	1.00		1.00	
> wM1	1.83 (1.02–3.29)	**0.042**	1.01 (0.52–1.95)	0.987

NOTE: ADC, adenocarcinoma; SCC, squamous cell carcinoma; CI, confidence interval; HR, hazard ratio.

^a^Bold values represented statistical significance (*P* < 0.05).

^b^The variables without significant HR in univariate analysis were not included in the multivariate analysis.

### Phosphorylation of Rab37 by PKCα abrogates Rab37 GTP binding state

**Figure 2:**
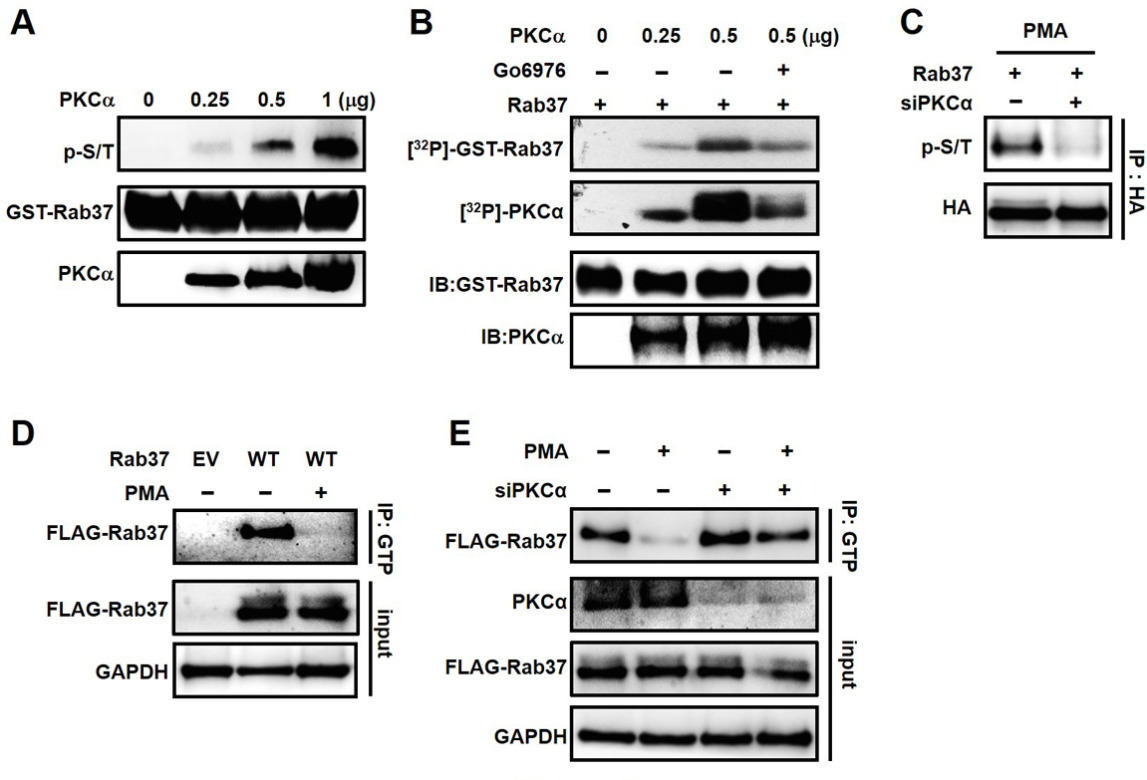
Rab37 phosphorylation by PKCα inhibited GTP binding ability (**A**) *In vitro* kinase assay was performed by incubating recombinant PKCα with purified GST-Rab37. The resulting mixtures were subjected to immunoblotting with antibodies against p-S/T proteins, Rab37 and PKCα. (**B**) Purified GST-Rab37 was incubated with [γ-^32^P]ATP and recombinant PKCα in the presence or absence of PKCα inhibitor Go6976. The autophosphorylation signals of PKCα served as a positive control in our *in vitro* kinase assay. (**C**) HA-Rab37 expressing PC-14 cells were transfected with non-specific siRNA (designated “-”) or siRNA mix against PKCα (designated “+”) for 48 hours. After treatment of PMA for 30 minutes, the cell lysates were prepared for IP with anti-HA antibody and followed by immunoblotting analysis with anti-p-S/T and -HA antibodies. (**D** and **E**) The treatment of PMA (D) and siRNA targeting PKCα (E) were the same as (C). The cell lysates were IP with GTP-conjugated beads followed by immunoblotting analysis with indicated antibodies.

Little is known about Rab37 phosphorylation in epithelial cells. We thus investigated whether Rab37 was phosphorylated in lung cancer cells by transfecting PC-14 cells with an empty vector (EV) or a plasmid carrying HA-tagged Rab37 wild-type (WT) insert. The phosphorylation of Rab37 was detected on the serine/threonine residue(s) (Supplementary Figure 1B). Further, *in vitro* kinase assay was performed to verify whether PKCα was responsible for phosphorylation of Rab37. As shown in Figure 2, phosphorylation levels of GST-Rab37 was enhanced with increasing PKCα activity (Figure 2A), whereas blocking PKCα activity by Go6976 inhibited the incorporation of [g-^32^p] ATP into GST-Rab37 (Figure 2B). These results indicated that PKCα is capable of phosphorylating Rab37 *in vitro*.

Notably, an increased *in vitro* PKCα kinase activity was observed as evident by autophosphorylated signals of PKCα (Figure 2B). To test whether PKCα phosphorylates Rab37 in lung cancer cell-based studies, we first detected the PKCα activity by measuring the phospho-substrates of PKCα in cells with overexpression of PKCα (Supplementary Figure 2). Next, we used PMA, a potent activator of PKCα, to stimulate Rab37 phosphorylation in PC-14 cells stably expressing WT-Rab37. PMA induced Rab37 phosphorylation on serine/threonine (p-S/T) residue(s), and the phosphorylated signal of Rab37 was inhibited by knockdown of PKCα (Figure 2C). Interestingly, PMA treatment abrogated Rab37 GTP binding as analyzed by immunoprecipitation (IP) with GTP-Sepharose affinity beads followed by immunoblotting analysis (Figure 2D). Notably, PKCα knockdown dramatically restored Rab37 GTP binding upon PMA treatment, suggesting that PKCα-mediated phosphorylation dampened the GTP-bound active state of Rab37 (Figure 2E). Taken together, these results suggested that PKCα-mediated Rab37 phosphorylation leads to reduction in GTP-bound Rab37.

### PKCα abolishes Rab37-mediated exocytosis function and metastasis suppression activity

**Figure 3:**
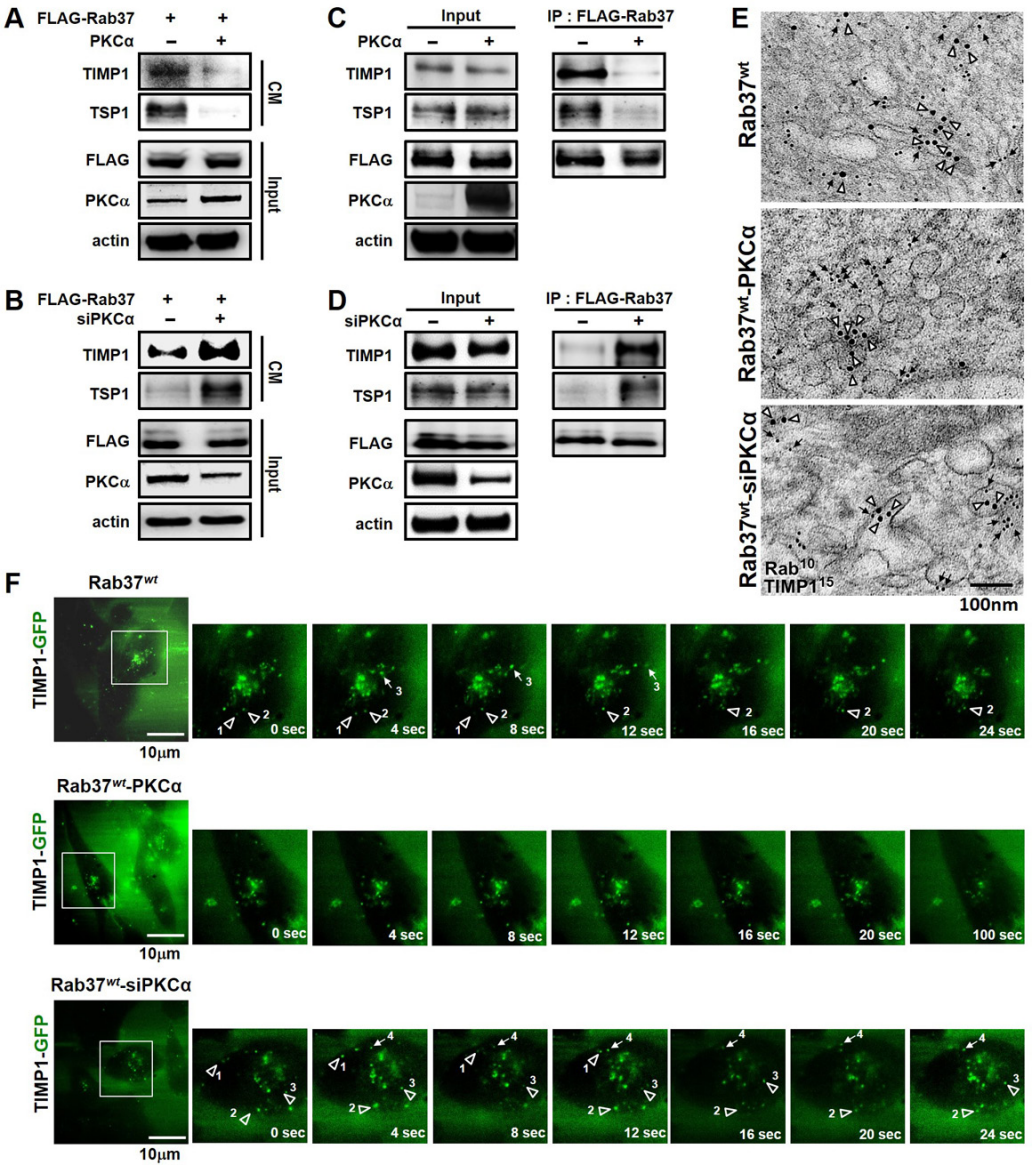
PKCα expression impaired Rab37-mediated exocytotic transport of TIMP1 and TSP1 (**A**) PC-14 cells stably expressing FLAG-tagged Rab37 (FLAG-Rab37) were transfected with empty or PKCα-expressing vectors. The cell conditioned medium (CM) was harvested and subjected to immunoblotting. (**B**) siRNA against PKCα was introduced into cells stably expressing FLAG-Rab37, the TIMP1 and TSP1 levels in CM was analyzed by immunoblotting. (**C** and **D**) Cells stably expressing FLAG-Rab37 were transfected with expressing vector (C) or siRNA oligos of PKCα (D). Vesicles were isolated by centrifugations and subjected to IP with anti-FLAG antibody followed by blotting for FLAG-Rab37 and endogenous TIMP1 and TSP1. (**E**) Cells stably expressing FLAG-Rab37*wt* were transfected with empty (upper) or PKCα-expressing vector (middle) or siRNA against PKCα (lower). The co-localization of TIMP1 (15 nm of gold, triangle) and Rab37 (10 nm of gold, arrow) in vesicles was observed in immuno-EM images. Scale bars 100 nm. (**F**) Selected frames from time-lapse movies of TIMP1 trafficking in cells expressing FLAG-Rab37*wt*, and GFP-TIMP1 together with overexpression (Rab37*wt*-PKCα) or knockdown (Rab37*wt*-siPKCα) of PKCα were shown. The numbers labeled horizontal (arrow) or vertical (triangle) movement of GFP-TIMP1. Enlarged images of the boxed areas from time-lapse movies with time intervals in seconds shown. Scale bars 10 µm.

To further characterize the effects of PKCα-mediated Rab37 phosphorylation on Rab37-regulated cargo secretion, we measured the amounts of Rab37-mediated secretion of cargos, TIMP1 and TSP1, in the conditioned medium (CM) from WT-Rab37-stable PC-14 cell line with transient PKCα-overexpression or knockdown. Notably, lower amounts of TIMP1 and TSP1 were observed in the CM from PKCα overexpressing cells (Figure 3A), while knockdown of PKCα increased TIMP1 and TSP1 level in CM (Figure 3B). These data were consistent with the model predicting the suppression of Rab37-mediated cargo secretion by PKCα. To further determine the mechanism for the suppression of Rab37-mediated cargo secretion by PKCα, FLAG-tagged WT-Rab37-specific vesicles were isolated and analyzed for levels of TIMP1 and TSP1. The results showed that TIMP1 and TSP1 were associated with Rab37-containing vesicle fractions and the association was abrogated by overexpressing PKCα (Figure 3C). In contrast, siRNA-knockdown of PKCα expression increased the association of TIMP1 and TSP1 with Rab37 in the same vesicle fractions (Figure 3D).

Furthermore, we performed immuno-electron microscopy (immuno-EM) assay for ultrastructural localization of Rab37 and TIMP1 and found that Rab37 and TIMP1 localized on the same vesicles (47%, upper; Figure 3E and Supplementary Figure 3A), while there was a marked reduction of colocalization of Rab37 and TIMP1 in PKCα-overexpressing cells where most Rab37 proteins were dissociated from the vesicle membranes and scattered with cytoplasm (18%, middle; Figure 3E and Supplementary Figure 3A). The reduction in Rab37 and TIMP1 colocalization was recovered in cells with PKCα knockdown (52%, lower; Figure 3E and Supplementary Figure 3A). Notably, the fractionation assay showed that WT-Rab37, but not phospho-deficient alanine substitution mutant at threonine-172 (T172A), associated with less membrane fraction upon activation of PKCα by PMA treatment (Supplementary Figure 3B). Additional mass spectrometry analysis and site-direct mutagenesis experiments to identify the Rab37 phosphorylation are described below.

Finally, the regulation of TIMP1 trafficking dynamics by Rab37 was analyzed by using total internal reflection fluorescence (TIRF) microscopy, which allowed us to monitor real-time images of fluorescently labeled vesicles located in close proximity to the plasma membrane (upper, Figure 3F; Supplementary Movie 1). Overexpression of PKCα dramatically attenuated Rab37-mediated TIMP1 exocytotic transport in WT-Rab37 cells (middle, Figure 3F; Supplementary Movie 2), whereas the TIMP1-containing vesicles trafficking events were more in PKCα-silenced WT-Rab37 cells than that in control WT-Rab37 cells (lower; Figure 3F; Supplementary Movie 3). These data therefore supported a mechanism by which PKCα blocks Rab37-mediated cargos secretion by facilitating the dissociation of phosphorylated Rab37 from its targeting vesicles.

### PKCα phosphorylates Rab37 on threonine-172

**Figure 4:**
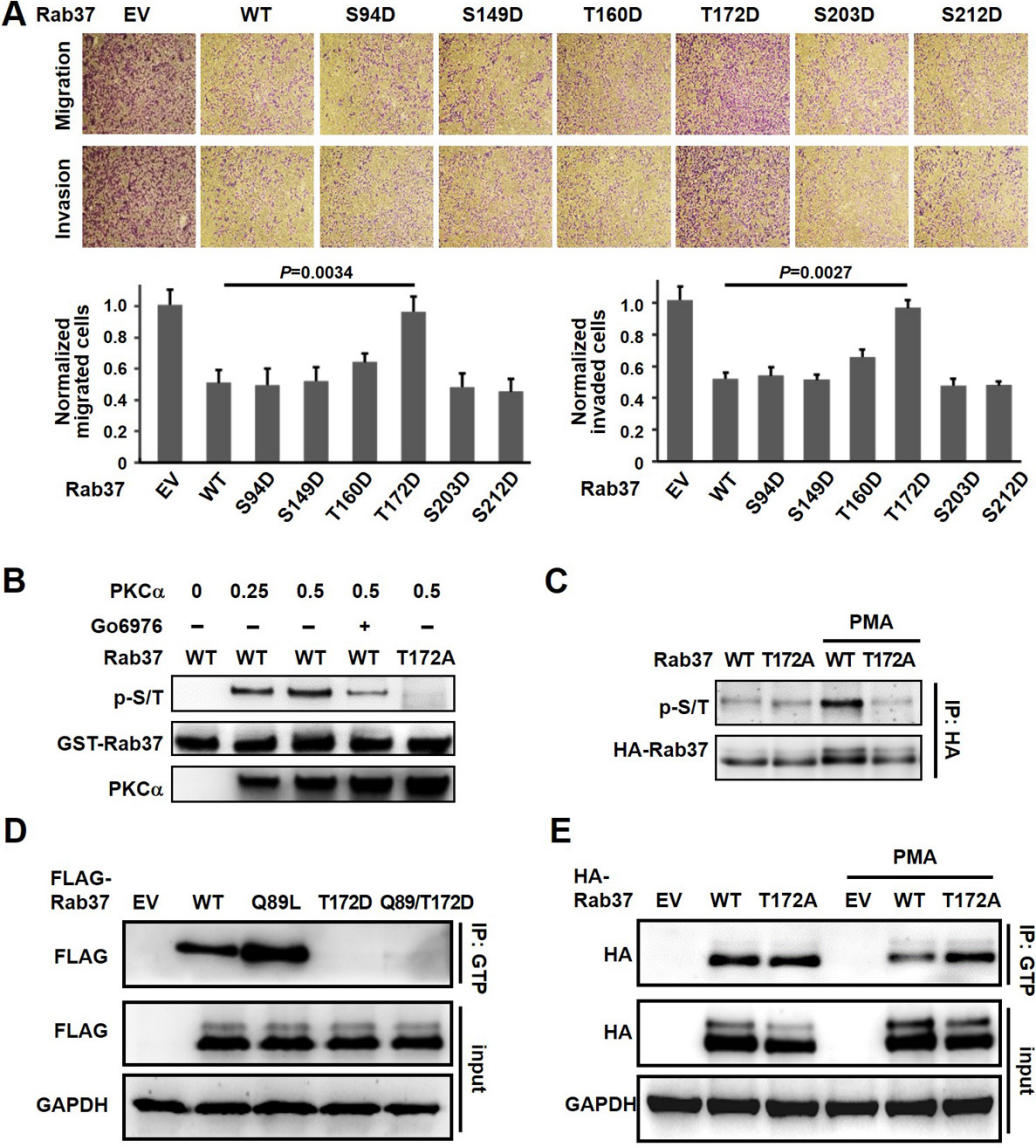
PKCα phosphorylated Rab37 at T172 and inhibited GTP binging ability (**A**) PC-14 cells were transfected with various Rab37 phospho-mimetic D mutants followed by analysis of cell migration and invasion abilities (upper). The quantitative results were determined by two-tailed Student’s *t-*test (lower). Data represented mean ± SD. (*n =* 3). Original magnification: 100X. (**B**) Recombinant PKCα and purified WT or GST-tagged Rab37-T172A were used in an *in vitro* kinase assay in the presence or absence of Go6976. The phosphorylation signal of Rab37 was analyzed by immunoblotting. (**C**) PC-14 cells expressing HA-tagged WT or T172A mutant of Rab37 protein were treated with PMA. The lysates were collected for IP with anti-HA antibody and immunocomplexes were blotted for p-S/T proteins and HA-tagged Rab37. (**D**) Lysates from cell expressing various FLAG-tagged Rab37 mutants were subjected to GTP binding assay. (**E**). EV, WT or T172A mutant of HA-tagged Rab37 were transfected into PC-14 cells, the lysates were used for GTP binding assay following PMA treatment.

To identify the phosphorylation site(s) on Rab37 by PKCα, an *in vitro* kinase assay with recombinant PKCα and purified GST-Rab37 was performed. Mass spectrometry analysis of the phosphorylated Rab37 after *in vitro* PKCα kinase reaction identified six potential sites on Rab37, designated as S94, S149, T160, T172, S203 and S212 that may be phosphorylated by PKCα. The phospho-mimetic aspartate (D) substitution mutants of these six candidate sites were constructed. Since Rab37 acts as a cell motility suppressor, the effects of these mutants on cell motility were then determined by transwell migration and invasion assays. Among the mutants, only the replacement of threonine-172 with aspartate T172D showed significant promotion of cell migration and invasion (Figure 4A). We also performed cell migration and invasion assays using Rab37 mutant constructs with the six potential residues mutated to alanine (A). These phospho-deficient mutants displayed similar migration and invasion abilities compared with WT-Rab37 (Supplementary Figure 4). These data suggested that PKCα may phosphorylate Rab37 at T172 to alter the Rab37-mediated functions. We then performed *in vitro* kinase assay to further confirm PKCα phosphorylation of Rab37 at T172. The p-S/T signal disappeared when GST-Rab37-T172A was used as the substrate of PKCα or in the presence of PKCα inhibitor Go6976 (Figure 4B). Moreover, PMA-induced Rab37 phosphorylation was inhibited in Rab37-T172A-expressing PC-14 cells (Figure 4C). These *in vitro* and cell-based results indicated that T172 of Rab37 is phosphorylated by PKCα.

### Threonine-172 phosphorylation disrupts Rab37 GTP binding

Since PKCα-mediated Rab37 phosphorylation reduced Rab37 in GTP-bound form (Figure 2) and impaired Rab37-mediated exocytic function (Figure 3), we therefore speculated whether PKCα-mediated T172 phosphorylation of Rab37 inhibited GTP binding ability of Rab37. As shown in Figure 4D, GTP-bound active glutamine-to-leucine of Rab37 (Q89L-Rab37) mutant displayed a significant increase in GTP binding compared to WT-Rab37. Interestingly, both T172D-Rab37 mutant and Q89L/T172D-Rab37 double mutant reduced the level of GTP-bound active Rab37 (Figure 4D), suggesting that T172 phosphorylation inhibited the activity of Rab37 via reducing the abundance of GTP-bound Rab37. Moreover, the amount of GTP-bound Rab37 in WT-Rab37 cells was diminished upon PMA treatment, whereas the GTP-bound form of phospho-deficient mutant T172A was not affected by PMA stimulation (Figure 4E, lanes 5 and 6). Collectively, our results suggested that PKCα-mediated Rab37 phosphorylation at T172 attenuates Rab37 activity through reduction in GTP-bound state.

### Phosphorylation of Rab37 on threonine-172 by PKCα abolishes its colocalization with TIMP1, resulting in loss of its motility suppressive function

**Figure 5:**
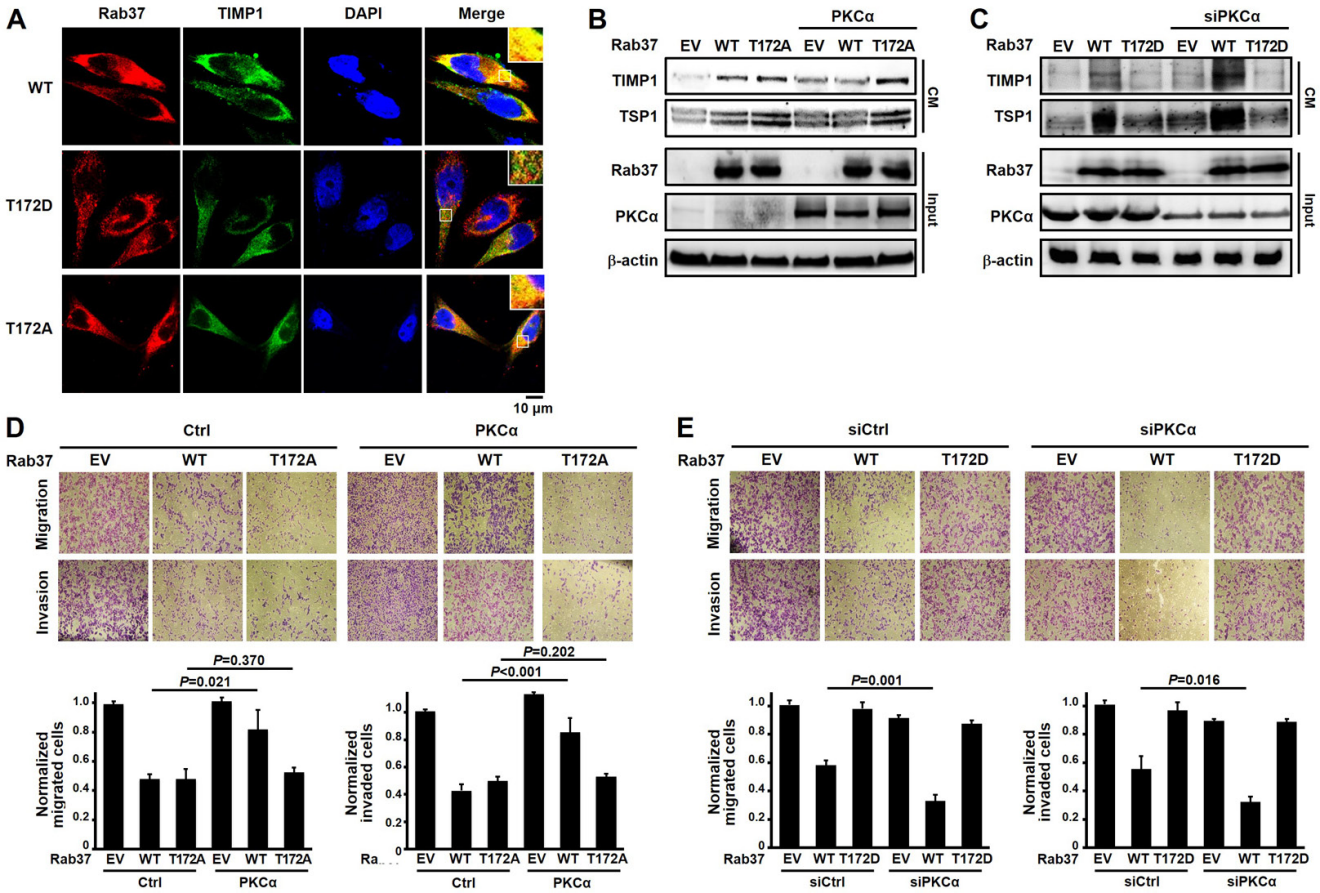
PKCα-mediated T172 phosphorylation of Rab37 was critical for modulating Rab37 activity (**A**) Confocal microscopic images of Rab37 (red), TIMP1 (green) and nucleus staining (blue) in WT-, T172D- and T172A-Rab37 expressing PC-14 cells. Enlarged images shown in insets of the merge panel. Scale bars, 10 μm. (**B** and **C**) CM were collected from PKCα-overexpressing (B) or -silencing (C) cells, which expressed T172A- or T172D-Rab37, respectively. The TIMP1 and TSP1 levels were analyzed by immunoblotting. (**D** and **E**) the manipulation of PKCα and mutant Rab37 in cells used in (B and C) were subjected to cell migration and invasion assay (upper) and quantitative analyses (lower). Data represented mean ± SD. (*n =* 3). Original magnification: 100X.

Since PKCα-mediated phosphorylation disrupted exocytic trafficking of TIMP1 and TSP1 by Rab37 (Figure 3) and reduced the GTP-bound state and membrane bound form of Rab37 (Figures 2 and 4), we further interrogated the cellular localization of Rab37 and its cargo TIMP1 in Rab37-WT, Rab37-T172D and Rab37-T172A cells. Indeed, ectopically expressed Rab37-WT or Rab37-T172A co-localized with TIMP1, whereas Rab37-T172D displayed distinct cellular distribution from TIMP1 in PC-14 cells (Figure 5A, merged panel). Accordingly, PKCα expression markedly decreased CM concentration of TIMP1 and TSP1 in Rab37-WT cells, but not in Rab37-T172A cells (Figure 5B). In contrast, lower CM level of TIMP1 and TSP1 were observed in cells expressing T172D mutant. Note that siRNA-mediated knockdown of PKCα significantly increased the CM concentration of TIMP1 and TSP1 in Rab37-WT cells. In addition, change in PKCα level did not alter the exocytic trafficking activity in the Rab37-T172D mutant-expressing cells (Figure 5C). Furthermore, overexpression of PKCα could not further interfere with the regulation on cell motility in the T172A cells (Figure 5D). Accordingly, silencing PKCα also could not lead to additional enhancement of cell migration in the T172D-expressing cells (Figure 5E). Of note, the amount of secreted TIMP1 and TSP1 level in CM from Rab-T172A and -T172D cells were consistent with the scenario that Rab37 phosphorylation at T172 attenuated cargo secretion and thus impaired Rab37-mediated suppression of cell migration and invasion (Figure 5). Similar results of inhibitory effects of PKCα-mediated T172 phosphorylation of Rab37 on cellular distribution, Rab37-mediated TIMP1 and TSP1 secretion and motility suppression were observed in another lung cancer cell line A549 (Supplementary Figure 5). These results indicated that PKCα-mediated T172 phosphorylation of Rab37 eliminates TIMP1 secretion and thus promotes cell motility.

### Phospho-mimetic mutant of Rab37 on threonine-172 promotes metastasis *in vivo*

**Figure 6:**
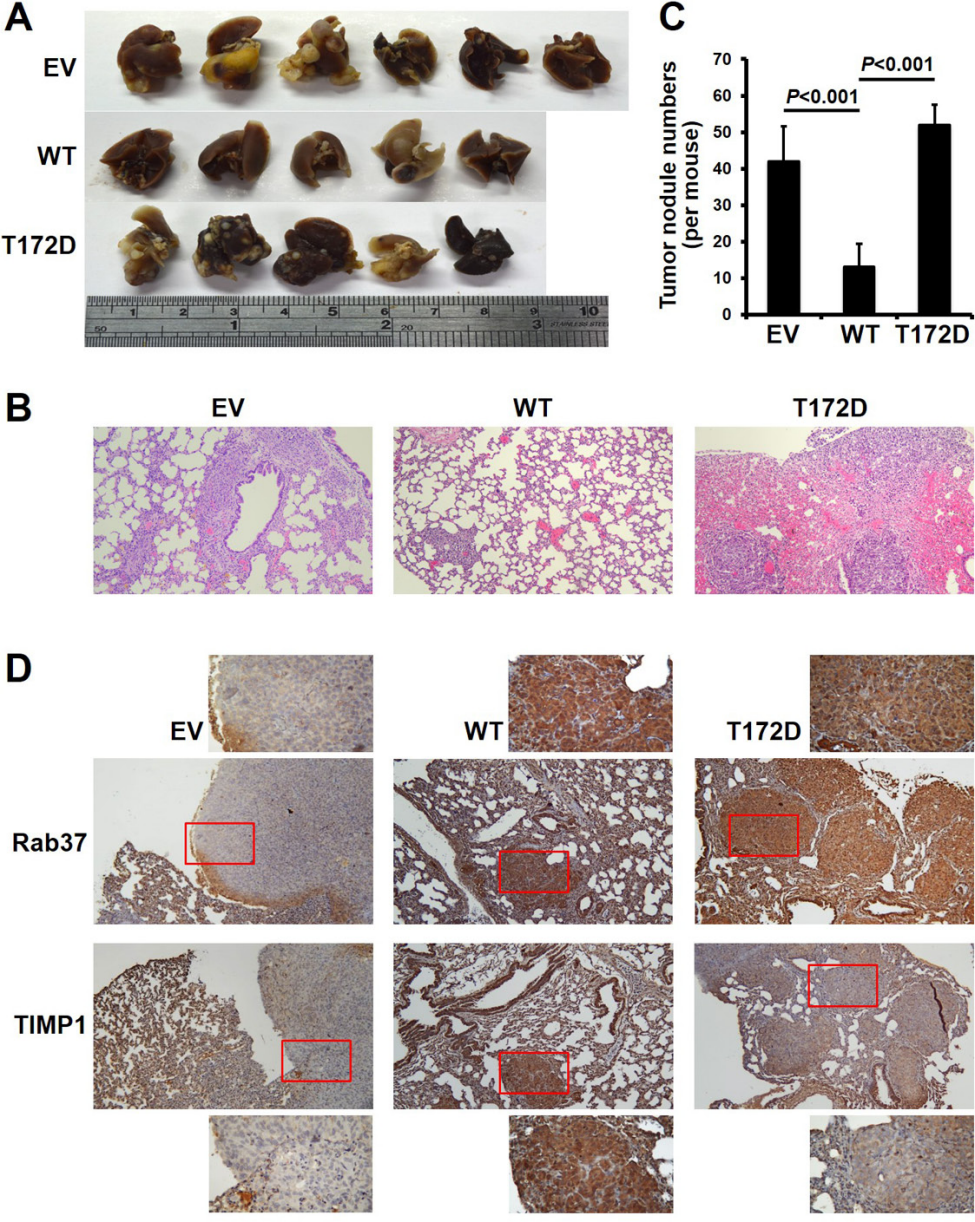
Phospho-mimetic T172D mutant of Rab37 lost its metastasis suppression ability *in vivo* (**A**) The lung images from nude mice 6 weeks after injection of PC-14 cells stably expressing EV-, WT- or T172D-Rab37 *via* the tail vein. (**B**) and (**C**) H&E staining shows more lung colonization and bigger tumor mass in EV control and T172D groups compared with WT group (B) (Original magnification: 200×). Numbers of tumor nodules are shown in the histogram plot (C). *P*-values were calculated by two-tailed Student’s *t-*test. Data represented mean ± SD. (*n =* 5–6 mice/group). (**D**) Lung tissues from EV-, WT- or T172D-injected nude mice were collected 6 weeks post-injection and subjected to immunohistochemistry to examine the expression of Rab37 and TIMP1. Enlarged images of insets are shown (Original magnification: 200×).

The inhibition of Rab37 activity by PKCα-mediated phosphorylation in cell-based model prompted us to investigate whether T172 phosphorylation of Rab37 impaired metastasis suppression in animals. We injected EV control, WT- and T172D-Rab37 expressing cells into the tail vein of nude mice. Consistent with our findings in cell model, T172D-Rab37 cells markedly enhanced lung metastases in injected mice compared with mice injected with WT-Rab37 cells (Figure 6A–6C). Importantly, when the concordant expression of WT-Rab37 and TIMP1 was confirmed *in vivo*, T172D-derived lung metastases showed similarly high Rab37 expression but lower TIMP1 staining as compared to that in WT-Rab37-derived lung metastases (Figure 6D). These *in vivo* results further support the notion that Rab37 phosphorylation at T172 inactivates the Rab37-mediated TIMP1 secretion and thus promotes tumor metastases.

## DISCUSSION

**Figure 7:**
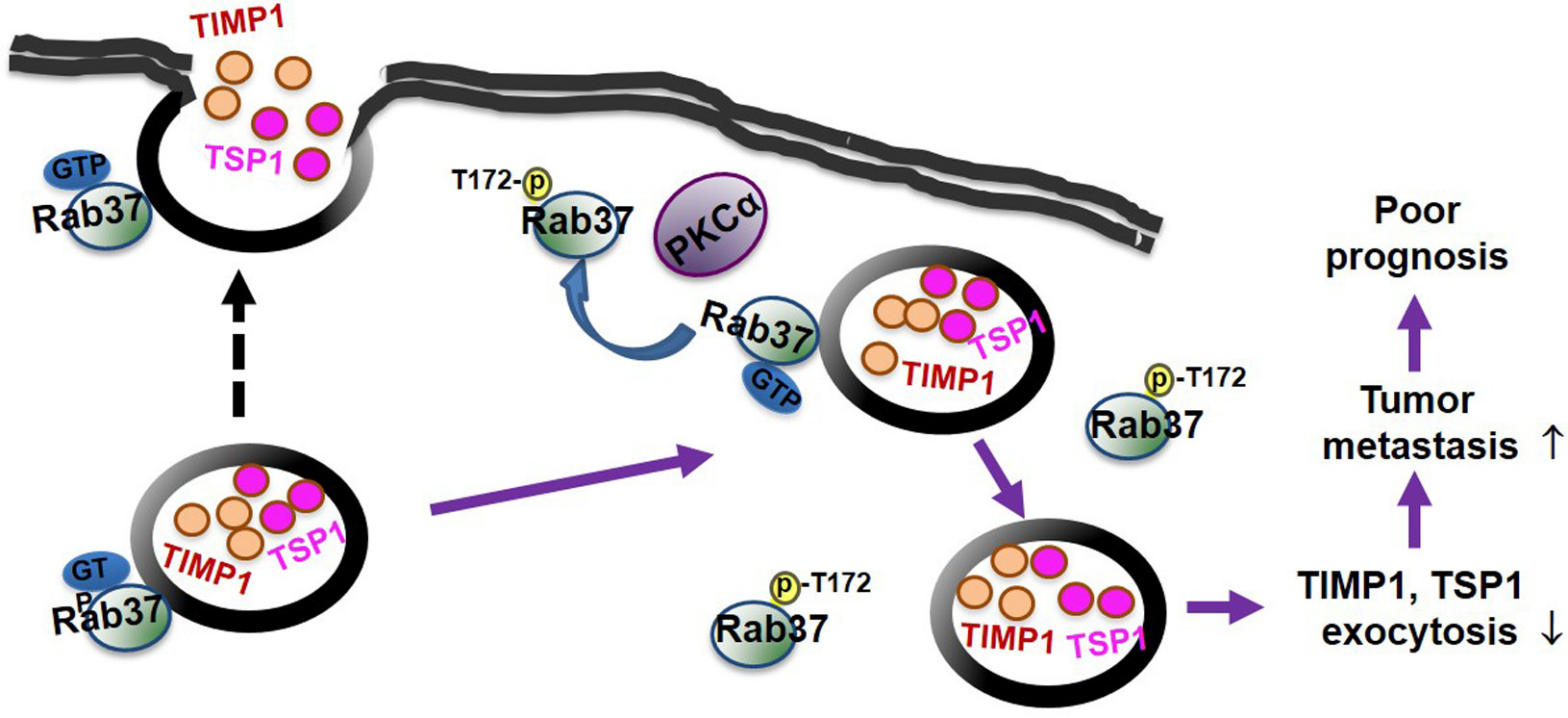
A schematic diagram illustrates that PKCα-mediated Rab37 phosphorylation inhibits the GTP-binding and exocytosis function as well as the metastasis suppressive effects of Rab37 leading to tumor progression and poor prognosis of lung cancer patients

In this study, we identify a novel mechanism for repression of Rab37 activity by PKCα-mediated phosphorylation via interfering with its GTP binding state. We unravel a phosphorylation site on Rab37 at threonine-172, which is critical for regulation of Rab37 activities including GTP-bound conformation, exocytosis of the cell motility inhibitors TIMP1 and TSP1, and metastasis suppression *in vitro*, *in vivo* and clinically (Figure 7). From a clinical point of view, we identified a group of lung cancer patients with a profile of high PKCα, high Rab37 and low TIMP1 secretion, and this group of patients displayed poor prognosis (Figure 1 and Tables 1–2). The mechanism for phosphorylation of Rab37 by PKCα leading to malfunction of Rab37-mediated exocytosis of TIMP1 and aggressive metastasis is further supported by our cell and animal data (Figures 3–6). Our findings provide the molecular insight into how Rab37 activity is regulated by phosphorylation coupled to the GTP/GDP binding mechanism (Figures 2 and 4) with prognostic implications.

Several Rab GTPases are identified as targets for PKC isozymes such as Rab5a, Rab6 and Rab11 [20–22]. Phosphorylation of Rab5a by PKCε promotes Rac1 activation to facilitate actin remodeling [20]. Conventional PKC-mediated Rab11 and Rab6 phosphorylation results in impaired endosomal recycling and redistribution in cytosolic fraction, respectively [21, 22]. However, it remains unclear whether PKC-mediated phosphorylation regulates activities in vesicle trafficking and cargo transport of these Rabs. Our results demonstrated that phosphorylation of Rab37 by PKCα suppressed the exocytotic activity of cargos, TIMP1 and TSP1, mediated by Rab37 (Figures 3–5). Images from confocal microscopy and immuno-EM analysis illustrated dislocation of Rab37 from TIMP1-containing vesicles upon PKCα overexpression (Figure 5A and Figures 3A–3E). Importantly, this dislocation may result from reduction of active GTP-bound Rab37 (Figures 2D and E). In addition, our results indicated that T172 phosphorylation inhibited GTP-bound Rab37 state (Figure 4E), suggesting that T172 phosphorylation has a crucial role in regulating the GTP-bound Rab37 activity.

Interestingly, threonine-172 is located in the G5 box (ETSAK), a conserved region that is involved in the guanine-nucleotide binding (Supplementary Figure 6). This raises the possibility that T172 phosphorylation impairs GTP binding via electric repulsion or interference of GTP entry into the binding pocket of Rab37 spatiotemporally. In addition, Rab GTPase activity is determined by interaction with effector complex and GDP/GTP binding state, which is catalyzed by GTPase-activating proteins (GAP) and guanine nucleotide exchange factors (GEF) [23]. A glutamine-to-leucine mutation of Rab37 (Q89L) stabilizes Rab in an active conformation by prevention of GAP-mediated GTP hydrolysis [24]. Unexpectedly, overexpression of PKCα abrogated the suppression of Q89L-Rab37 active mutant on cell motility (Supplementary Figure 7) and T172 phosphorylation of Rab37 reduced the level of GTP-bound Q89L mutant (Figure 4D). We therefore speculated that T172 phosphorylation of Rab37 might affect the interaction with its cognate effectors. Indeed, studies have shown that phosphorylation of Rab4 by p34^cdc2^ prevents the association of Rab4 with endosomal membrane by dissociating the binding to membrane effector during the cell cycle [25, 26]. Rab8A phosphorylation on S111 is also observed to impair its binding to Rabin8, the GEF for Rab8A that triggers GDP exchange [27]. It will be interesting to identify the effector proteins and GEF for Rab37 to clarify this notion.

The downstream targets of PKC family as predicted by computational strategies commonly contain linear consensus motifs such as (S/T)X(K/R), (K/R)XX(S/T) or (L/R)X(S/T). Here we performed *in vitro* kinase assay with recombinant PKCα and purified Rab37 in conjunction with mass spectrometry analysis and site-direct mutagenesis assay to identify the T172 residue of Rab37 as the site that is phosphorylated by PKCα (Figures 2 and 4). However, T172 is not within a part of linear PKC consensus sequences. Notably, new strategies with advanced chemical biology, mass spectrometry and proteomics have revealed that many protein kinase substrates are phosphorylated on residues that are not within the software-predicted linear consensus motifs [28–30]. Instead, the phosphorylation sites recognized by kinases occur within a region that is created by structure-based non-linear consensus motif [31]. Thus, it is speculated that the sequence surrounding T172 on Rab37 may create a structure-based consensus motif. Further structure biology studies will clarify how PKCα recognizes sequence flanking T172 residue on Rab37.

PKCα has long been recognized as an oncoprotein in several types of cancer by regulating tumor growth and progression [32–36]. Both of our cell-based and animal studies revealed that phospho-mimetic mutant of Rab37 at T172, the substrate site of PKCα, profoundly enhanced cell motility and lung metastasis (Figures 4–6). These results link the oncogenic signaling of PKCα to attenuation of metastasis suppressive function of Rab37.

In conclusion, our study provides a new mechanistic insight into the regulation of Rab37 activity by PKCα-dependent phosphorylation leading to inhibition of Rab37-mediated exocytosis of anti-migratory factor/cargo and thus facilitating cell motility and cancer metastasis. Phosphorylation on T172 of Rab37 significantly suppresses its binding affinity for GTP. New diagnostic and therapeutic strategies based on detection of Rab37 phosphorylation and inhibitors against phosphorylated Rab37 could improve the survival in lung cancer patients.

## MATERIALS AND METHODS

### Cell culture and reagents

Human lung cancer cell line PC-14 was obtained from Dr. Pan-Chyr Yang (Department of Internal Medicine, Medical College, National Taiwan University, Taipei, Taiwan). A549 was purchased from ATCC. Stable expression of FLAG-tagged WT-, active Q89L- and inactive T43N-Rab37 PC-14 cells were generated as described previously [18]. The small interfering RNAs pool containing 5′-UAAGGAACCACAAGCAGUA-3′, 5′-UUAUAGGGAUCUGAAGUUA-3′, 5′-GAAGGGUUCUCGUAUGUCA-3′, 5′-UCACUGCUCUAUGGACUUA-3′ (Dharmacon, DE, USA) was used to knockdown PKCα. Phorbol 12-myristate 13-acetate (PMA) and conventional PKC inhibitor (Go6976) were from Sigma-Aldrich (St. Louis, MO, USA).

### Site-directed mutagenesis and *in vitro* kinase assay

Phospho-deficient and -mimetic mutants of Rab37 were generated by PCR-based site-directed mutagenesis. Serine (S) or threonine (T) was mutated to alanine (A) or to aspartate (D). The primer sequences used and PCR reactions are listed in Supplementary Table 1. All constructs were verified by sequencing. The resulting products were then transformed into *E.coli*. To analyze protein phosphorylation level, the GST-fused Rab37 proteins (Rab37-WT or Rab37-T172A) and recombinant PKCα (Millipore, MA, USA) were incubated with [γ-^32^P]ATP followed by autoradiography or by Western blot using antibody against p-S/T peptides (Supplementary Table 2).

### Vesicle isolation, immunoprecipitation and cellular fractionation

Vesicle isolation protocol was modified from Hendrix’s report [15]. In brief, cells (2 × 10^8^) were sonicated and supernatants were obtained by centrifugation (3,000 g for 10 min at 4°C) and vesicles were enriched from supernatants by high speed centrifugation (30,000 g for 60 min at 4°C) using a 40-Ti rotor (Beckman Coulter, CA, USA). The vesicles-containing solution (800 μg) was incubated with anti-Flag antibody to isolate Rab37-specific vesicles and the cargos in vesicles were analyzed by immunoblotting using indicated antibodies (Supplementary Table 2). For cellular fractionation, intact cells suspended in the lysis buffer (25 mM Hepes, pH6.5, 250 mM sucrose, 1mM EDTA and protease inhibitor cocktail) were pelleted by centrifugation (250 g for 10 min). The cytoplasmic (supernatant) and membrane (pellet) fractions were then obtained by centrifugation at 16000 g for 60 min at 4°C.

### Confocal, immuno-electron microscopy (immuno-EM), and total internal reflection fluorescence (TIRF) imaging

For confocal analysis, cells (1 × 10^5^) were fixed and then incubated with primary antibodies against Rab37 and TIMP1. After incubation with secondary fluorescent antibodies, the images were obtained using Olympus FV1000 confocal microscope. For immuno-EM analysis, primary antibodies and the conjugated 10 or 15 nm gold particles were listed in Supplementary Table 2. For TIRF analysis, cells expressing WT-Rab37 were transiently transfected with GFP-TIMP1 for 24 h before image analysis. To capture the Rab37 trafficking events, we tracked each vesicle trafficking distance with trackIT software (Olympus, Tokyo, Japan).

### Immunoblotting

Cells were lysed in 1× RIPA lysis buffer with a cocktail of antiproteases (Sigma-Aldrich, MO, USA), equal amounts of total cellular extracts were electrophoresed by SDS-PAGE and transferred on to a polyvinylidene difluoride membrane. Primary antibodies used for immunoblotting are list in Supplementary Table 2.

### Migration and invasion assays

For transwell migration and invasion assays, 1 × 10^5^ cells were seeded onto the upper chamber with or without Matrigel (Sigma-Aldrich, MO, USA) and cultured for 16 h. Cells attached on the reverse side of the membrane were then fixed and stained. Ten random views were photographed and quantified.

### Experimental metastasis assays *in vivo*

BALB/c nude mice were purchased from Laboratory Animal Center of National Cheng Kung University. All experimental procedures involving the use and care of mice were approved by the Institutional Animal Care and Use Committee of National Cheng Kung University (#104165). All protocols were performed according to the guidelines of “Law Concerning the Protection and Control of Animals” and “Animal Protection Act”. Mice were divided into three groups including control EV cells, WT-Rab37 cells, and T172D-Rab37 cells. Cells were resuspended in serum-free medium (2 × 10^6^ cells per 200 ml) and injected into BALB/c nude mice via the tail vein. Mice were sacrificed after 6 weeks and lung tissues were resected, fixed and embedded in paraffin for histological H&E and immunohistochemistry staining using indicated antibodies (Supplementary Table 2).

### Clinical samples and information

We recruited 158 lung cancer patients from National Cheng Kung University Hospital (NCKUH), Taiwan after obtaining appropriate institutional review and informed consent from patients. This study was approved by the Institutional Review Board of NCKUH (B-ER-103-369). All clinical samples used were conducted in accordance with the guidelines of the Declaration of Helsinki. Paraffin sections were collected for immunohistochemistry analysis. Overall survival was calculated from the day of surgery to the date of death or the last follow-up. The end of the follow-up period was defined as June 2016. The mean follow-up period was 64.8 months (range 9–103 months).

### Statistical analysis

Statistical analysis was performed by Student’s *t* test. Data shown were representatives of at least three independent experiments unless indicated otherwise. Pearson’s χ^2^ test was used to compare the correlation of between PKCα, TIMP1 and Rab37 expression levels and their association with clinicopatholgical parameters in lung cancer patients. Overall survival curves were calculated according to the Kaplan–Meier analysis. Cox regression comparison was performed to analyze the relative risk for poor outcome in patients. Data represented mean ± SD. *P* < 0.05 was considered to be statistically significant.

## SUPPLEMENTARY MATERIALS FIGURES, TABLES AND VIDEOS









## Abbreviations

TIMP1: tissue inhibitor of metalloproteinases 1
PKCα: protein kinase Cα
TSP1: thrombospondin-1
CIP: calf intestinal phosphatase
PMA: phorbol 12-myristate 13-acetate
CM: conditioned medium
EM: electron microscopy
TIRF: total internal reflection fluorescence
GAP: GTPase-activating proteins
GEF: guanine nucleotide exchange factors
