# Supplementary material for: Phosphorylation of Rab37 by protein kinase C alpha inhibits the exocytosis function and metastasis suppression activity of Rab37
Source: Oncotarget. 2017 Sep 18;8(65):108556–70. doi: 10.18632/oncotarget.20998 (PMC5752464; doi:10.18632/oncotarget.20998)
Supplement: Supplementary file 1 [file oncotarget-08-108556-s001.pdf]

Phosphorylation of Rab37 by protein kinase C alpha inhibits the exocytosis function and metastasis suppression activity of Rab37

SUPPLEMENTARY MATERIALS

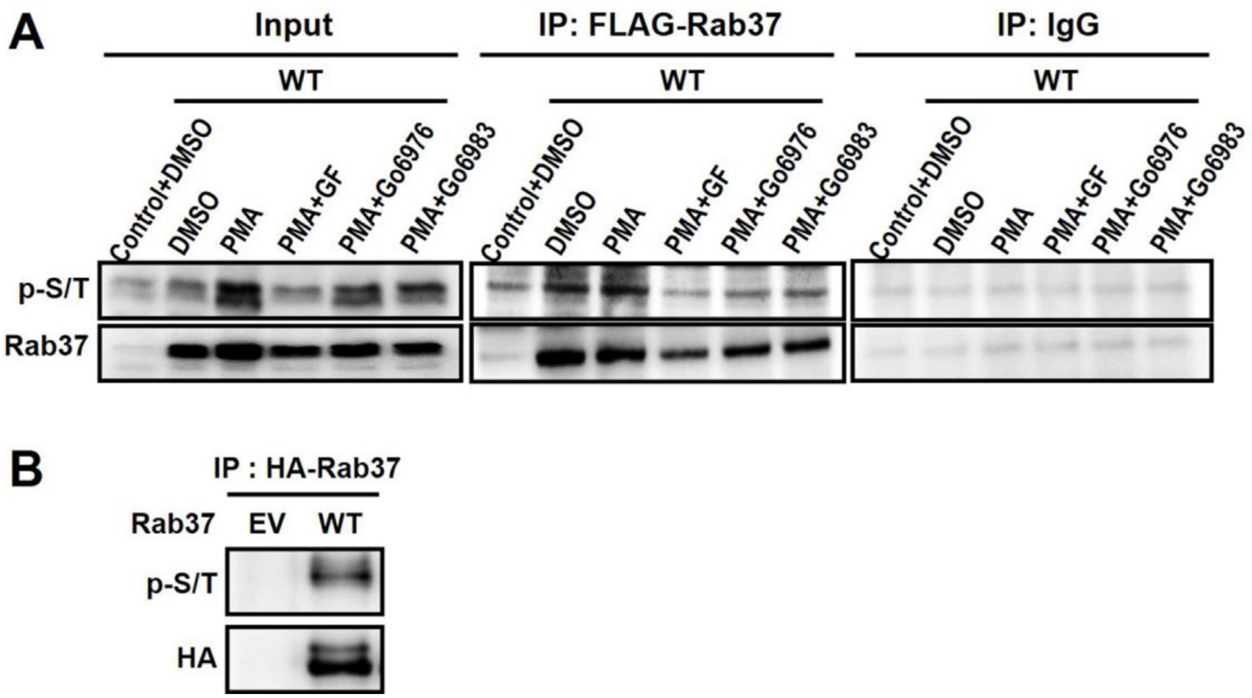

Supplementary Figure 1: PKC phosphorylates Rab37 in lung cancer cells. (**A**) PKC activator and inhibitors were used to examine Rab37 phosphorylation level in Rab37 stable expressed in PC-14 (WT) cells. PKC activator (PMA) was treated in WT cells for 30 min then to examine Rab37 phosphorylation level by immunoprecipitation-western blot (IP-WB). Pan-PKC inhibitor (GF109203x, GF), conventional and novel PKC inhibitor (Go6983), or conventional PKC inhibitor (Go6976) was treated in WT cells for 15 min after pre-treatment of PMA for 15 min to examine Rab37 phosphorylation level by IP-WB. The phosphorylation level of Rab37 was examined by phospho-serine/threonine (p-S/T) blots in IP: FLAG group. Cell lysates were collected to confirm Rab37 expression level in Input group. (**B**) Phosphorylation of Rab37 on serine/threonine residues. Lysates of PC-14 cells expressing HA-Rab37 protein were IP with anti-HA antibody and immunocomplex were blotted for p-S/T proteins and HA-tagged Rab37.

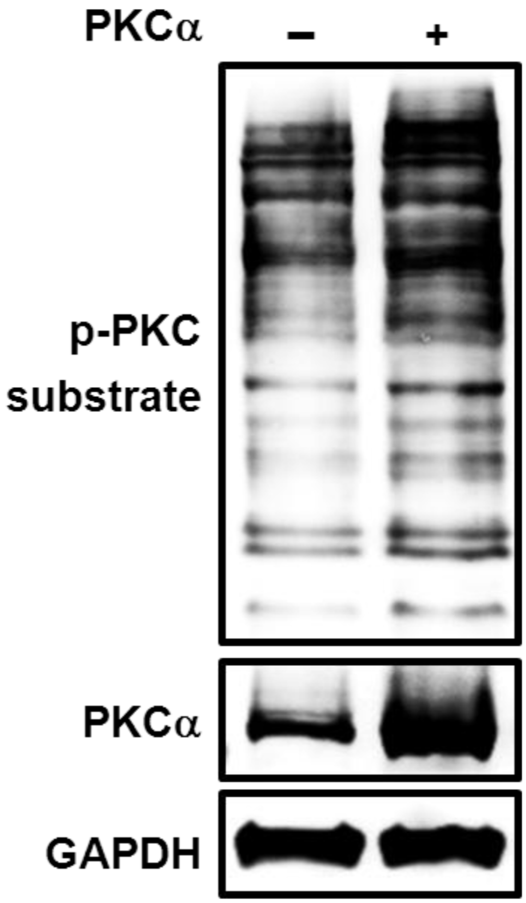

Supplementary Figure 2: PKCα activity in lung cancer cells. PC-14 cell lysates were prepared from empty vector control (−) or overexpression of PKCα (+) and then subjected to immunoblotting using antibodies specific for phospho-substrates of PKCα, PKCα and GAPDH.

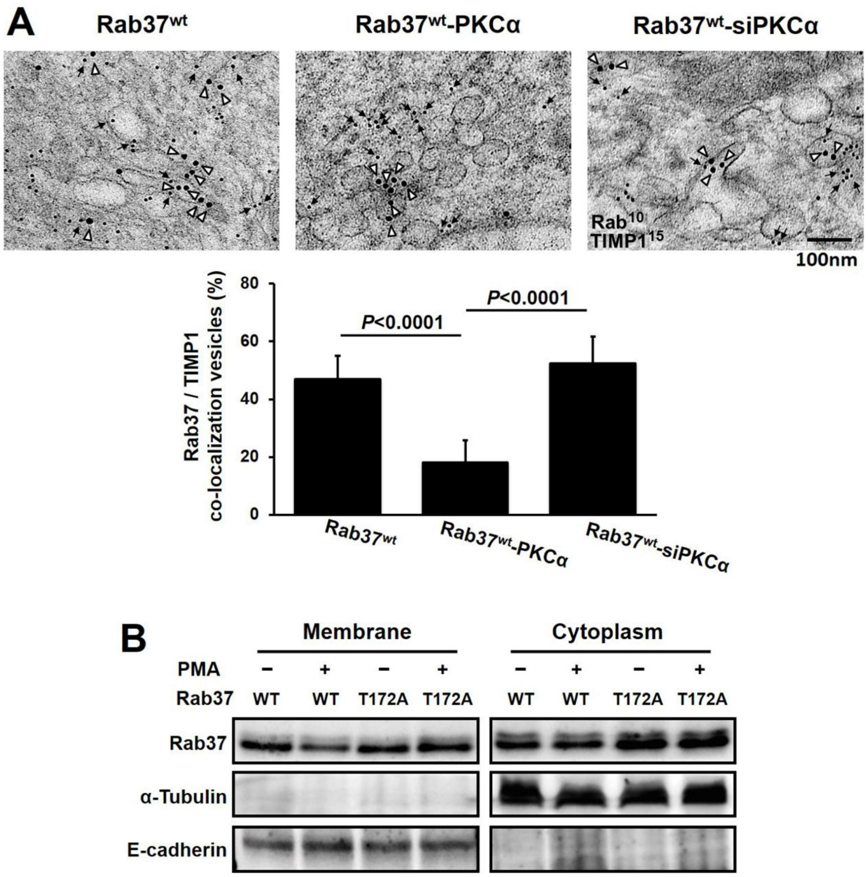

Supplementary Figure 3: Co-localization of Rab37 and TIMP1 in vesicles as well as membrane enrichment were abrogated by PKCα expression. (**A**) Cells stably expressing FLAG-Rab37*wt* were transfected with empty vector (left) or PKCα-expressing vector (middle) or siRNA against PKCα (right). The co-localization of TIMP1 (15 nm of gold, triangle) and Rab37 (10 nm of gold, arrow) in vesicles was observed in immuno-EM images. Scale bars 100 nm. Co-localization of Rab37 and TIMP1 in the same vesicle was quantified in 100 TIMP1-containing vesicles each in empty vector, PKCα-expressing or PKCα-silencing cells with Rab37 stably expression (low). The vesicles count is shown as a percentage in the graph. (**B**) PC-14 cells expressing HA-tagged WT or T172A mutant of Rab37 protein were treated with PMA. The cell pellets were harvested and membrane/cytoplasmic fractions were isolated for immunoblotting.

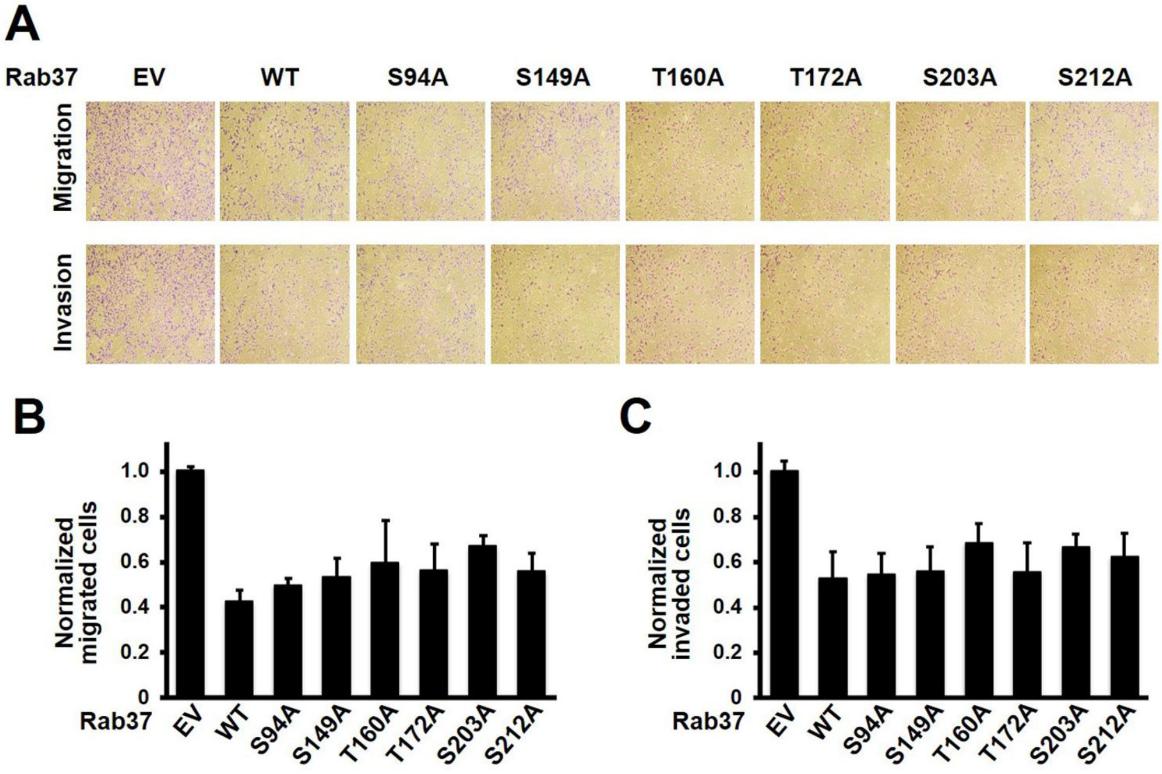

Supplementary Figure 4: Phospho-deficient mutants of Rab37 suppress cell motility. (**A**) PC-14 cells were transfected with EV-, WT-Rab37 and various Rab37 phospho-deficient alanine (A) mutants. The migration and invasion assay were performed for 24 hours. Original magnification: 100×. (**B** and **C**), cell migration and invasion abilities were analyzed. P-values were determined by two-tailed Student's *t*-test. Data represented mean ± SD ($n = 3$).

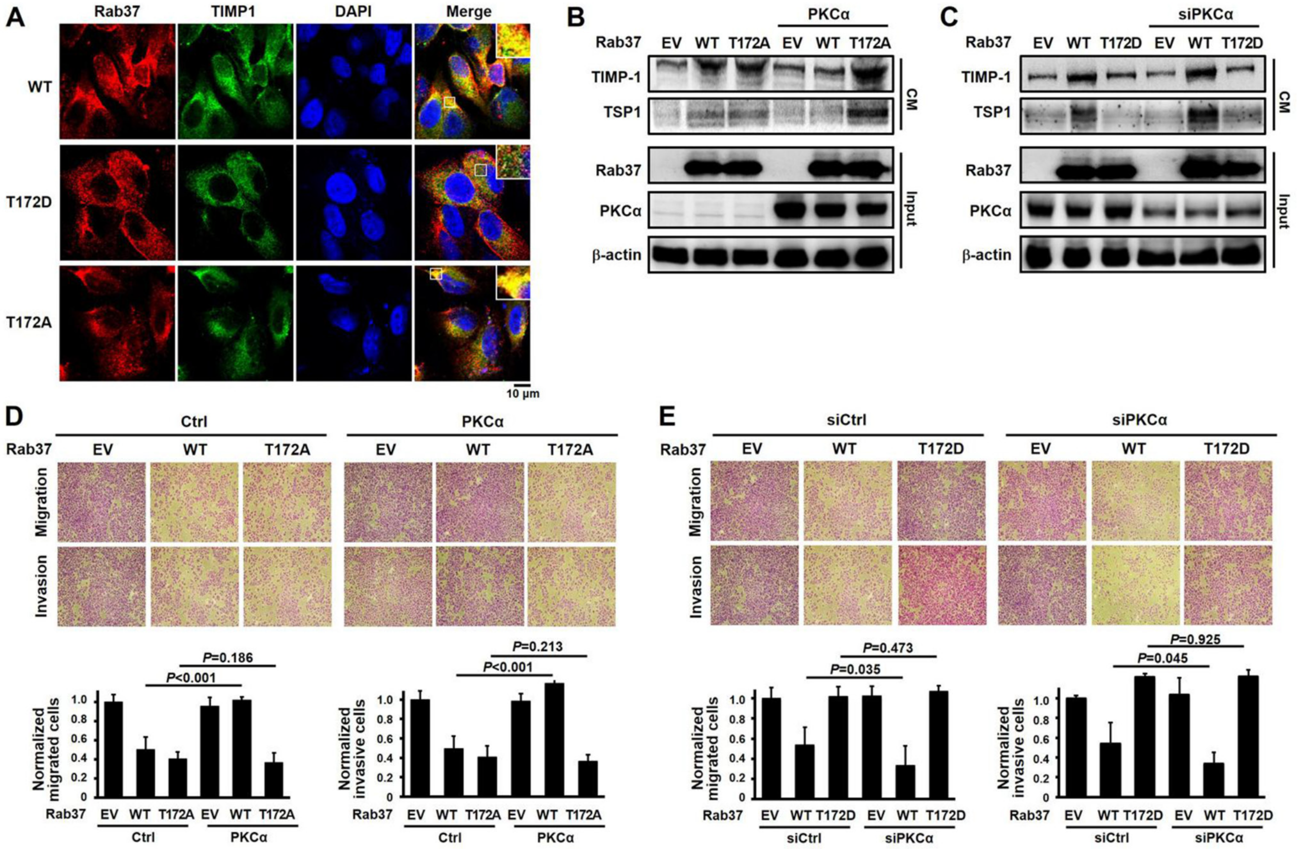

Supplementary Figure 5: PKCα-mediated Rab37 phosphorylation at T172 residue was critical for modulating Rab37 activity. (**A**) PC-14 cells were transfected with WT-, T172D- and T172A-Rab37 vectors. Confocal microscopic images of Rab37 (red), TIMP1 (green) and nucleus staining (blue) were shown. Enlarged images shown in insets of the merge panel. Scale bars, 10 μm. (B and D), conditioned media (CM) were collected from PKCα-expressing (**B**) or -silencing (**C**) PC-14 cells, which expressed WT-, T172A- or T172D-Rab37, respectively. The TIMP1 level in CM was analyzed by immunoblotting. (**D** and **E**), cells used in B and C with manipulation of PKCα and mutant Rab37 were subjected to cell migration and invasion assay (upper) and quantitative analyses (lower). Data represented mean ± SD (n = 3). Original magnification: 100×.

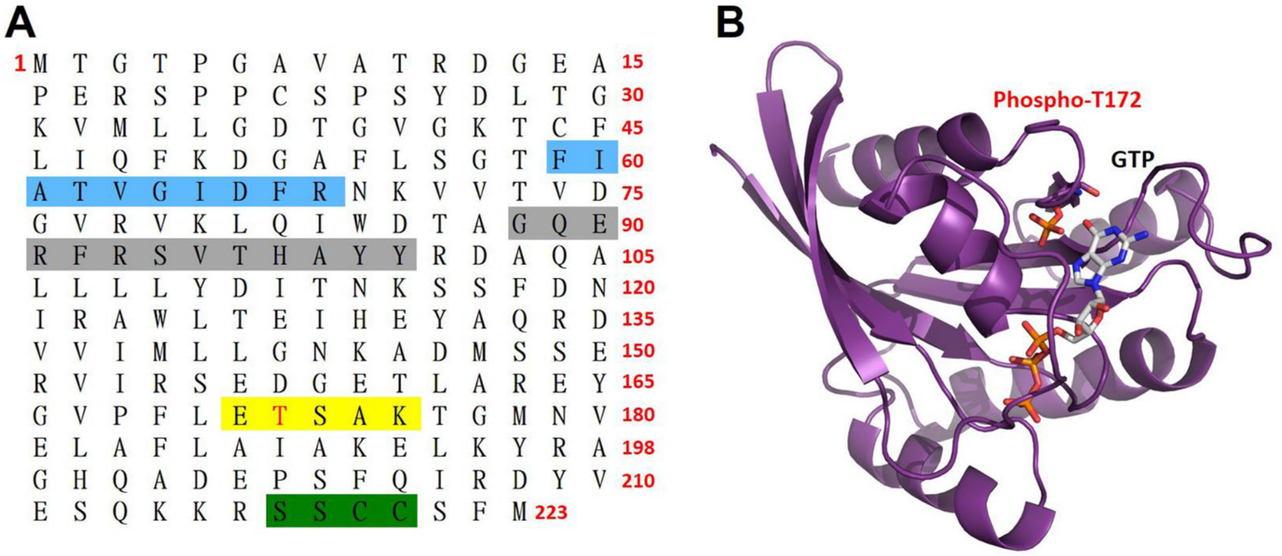

Supplementary Figure 6: Molecular simulation of threonine-172 residue phosphorylation of Rab37 and GTP binding. (**A**) Full-length amino acid sequence of Rab37 is shown. The conserved sequences of switch I and II regions among Rab family proteins are highlighted in blue and gray, respectively. C-terminal residues for prenylation are highlighted in green. G5 consensus motif including threonine-172 (red) is highlighted in yellow. (**B**) Mapping of phosphorylated Rab37 at threonine-172 and GTP binding within G5 motif.

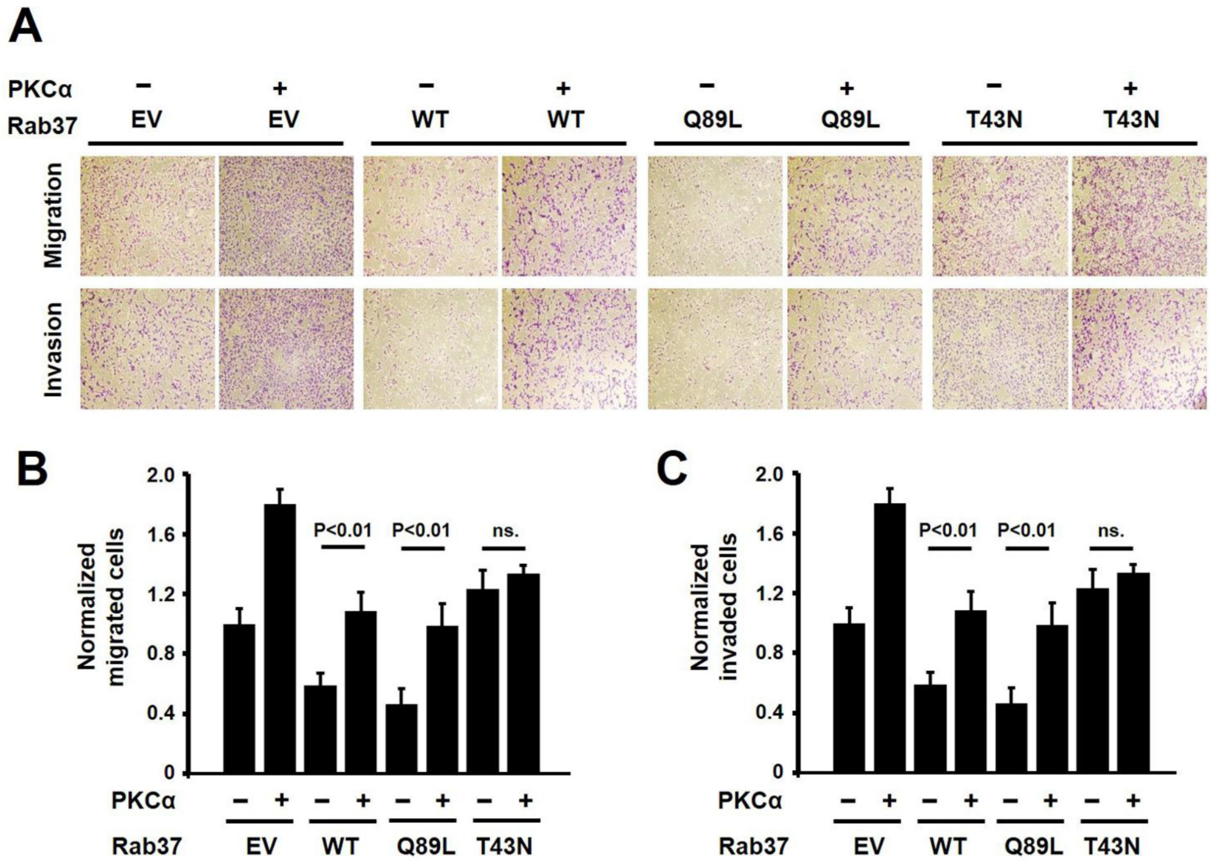

Supplementary Figure 7: PKCα abolished GTP-bound Rab37-mediated suppression of cell motility. (**A**) PKCα-expressing (+) or control (−) vectors were transfected into PC-14 cells with stable expression of WT-, Q89L- (actively mutant) or T43N- (inactively mutant) Rab37. The migration and invasion assay were performed for 24 hours. Original magnification: 100×. (**B** and **C**), cell migration and invasion abilities were analyzed. P-values were determined by two-tailed Student's *t*-test. Data represented mean ± SD (n = 3).

Suppplementary Movie 1: Time-lapse movie of TIRF images of Rab37-PC-14 cells expressing GFP-tagged TIMP1. See Suppplementary_Movie_1

Suppplementary Movie 2: Time-lapse movie of TIRF images of Rab37-PC-14 cells expressing GFP-tagged TIMP1 and PKCα expressing vector. See Suppplementary_Movie_2

Suppplementary Movie 3: Time-lapse movie of TIRF images of Rab37-PC-14 cells expressing GFP-tagged TIMP1 and RNAi ologos targeting PKCα. See Suppplementary_Movie_3

Supplementary Table 1: The primers used in the current study

Gene	Primer	Sequences (5'→3')	Application[a]	PCR size (bp)	Tm (°C)
Rab37 S94A	Forward	AA CGG TTC CGA GCC GTC ACC CAT	Site-direct mutagenesis	4425	60
	Reverse	ATG GGT GAC GGC TCG GAA CCG TT			
Rab37 S94D	Forward	CGG TTC CGA GAC GTC ACC	Site-direct mutagenesis	4425	60
	Reverse	GGT GAC GTC TCG GAA CCG			
Rab37 S149A	Forward	GAT ATG AGC GCC GAA AGA GTG	Site-direct mutagenesis	4425	60
	Reverse	CAC TCT TTC GGC GCT CAT ATC			
Rab37 S149D	Forward	GAT ATG AGC GAC GAA AGA GTG	Site-direct mutagenesis	4425	60
	Reverse	CAC TCT TTC GTC GCT CAT ATG			
Rab37 T160A	Forward	AGACGGAGAGGCCTTGGCCA	Site-direct mutagenesis	4425	60
	Reverse	TGGCCAAGGCCTCTCCGTCT			
Rab37 T160D	Forward	AGACGGAGAGGACTTGGCCA	Site-direct mutagenesis	4425	60
	Reverse	TGGCCAAGTCCTCTCCGTCT			
Rab37 T172A	Forward	TG GAG GCC AGC GCC AAG	Site-direct mutagenesis	4425	60
	Reverse	CTT GGC GCT GGC CTC CA			
Rab37 T172D	Forward	CTG GAG GAC AGC GCC AAG	Site-direct mutagenesis	4425	60
	Reverse	CTT GGC GCT GTC CTC CAG			
Rab37 S203A	Forward	ATGAGCCCGCATTCCAGATCC	Site-direct mutagenesis	4425	60
	Reverse	GGATCTGGAATGCGGGCTCAT			
Rab37 S203D	Forward	ATGAGCCCGACTTCCAGATCC	Site-direct mutagenesis	4425	60
	Reverse	GGATCTGGAAGTCGGGCTCAT			
Rab37 S212A	Forward	GAGACTATGTAGAGGCCCAGAAGAAGCG	Site-direct mutagenesis	4425	58
	Reverse	CGCTTCTTCTGGGCCTCTACATAGTCTC			
Rab37 S212D	Forward	GAGACTATGTAGAGGACCAGAAGAAGCGCTC	Site-direct mutagenesis	4425	58
	Reverse	GAGCGCTTCTTCTGGTCCTCTACATAGTCTC			

[a]For construction of Rab37 Q89/T172D mutant, we used Q89L-Rab37 as the PCR template and the primers of T172D in site-direct mutagenesis.

Supplementary Table 2: Antibodies and their reaction conditions used in the present study

Target	K.D.	Source	Application[a]	Dilution	Source	Catalog No.
b-actin	42	Mouse	WB	1:5000	Abcam	ab3280
DAPI	--[b]	--[c]	IF	1:5000	Sigma Aldrich	D8417
Flag	--[b]	Rabbit	WB	1:1000	Santa Cruz	sc-807
Flag	--[b]	Mouse	IP	1:1000	Sigma	F1804
HA	--[b]	Rabbit	WB	1:1000	Genetex	GTX29110
Rab37	30	Mouse	WB, IF, IHC, EM	1:500; 1:200; 1:400; 1:20	LTK BioLaboratories	Homemade
p-S/T	--[b]	Rabbit	WB	1:1000	ECM bioscience	PP2551
IgG	150	Mouse	IP	1:1000	Millipore	12-371
PKCα	72	Rabbit	WB, IHC	1:1000; 1:400	Abcam	Ab32376
phospho-substrates of PKCα	--[b]	Rabbit	WB, IP	1:1000	Cell Signaling Techmology	#2261
TIMP1	34	Rabbit	WB, IF, IP, EM	1:500; 1:200; 1:500; 1:50	Epitomic	2109-S
TIMP1	34	Rabbit	IHC	1:100	Spring Bioscience	E3364
Fluor 488	--[b]	Rabbit	IF	1:1000	Invitrogen	A11008
Fluor 546	--[b]	Rabbit	IF	1:1000	Invitrogen	A11035
Fluor 555	--[b]	Mouse	IF	1:1000	Invitrogen	A21422
15nm immune- Gold	--[b]	Rabbit	Immuno-EM	1:10	BBInternational	EM GAR15
10nm immune- Gold	--[b]	Mouse	Immuno-EM	1:10	Abcam	Ab27241

[a]WB: western blot, IF: immunofluorescence, IP: immunoprecipitation, IHC: immunohistochemistry, EM: electron microscope.

[b]Molecular weight is variable.

[c] Used for nuclear staining.
